# Einsatz von Maschinellem Lernen für die Vorhersage von Stress am Beispiel der Logistik

**DOI:** 10.1007/s41449-021-00263-w

**Published:** 2021-07-13

**Authors:** Hermann Foot, Benedikt Mättig, Michael Fiolka, Tim Grylewicz, Michael ten Hompel, Veronika Kretschmer

**Affiliations:** 1 Fraunhofer-Institut für Materialfluss und Logistik IML, Joseph-von-Fraunhofer-Str. 2–4, 44227 Dortmund, Deutschland; 2 Lehrstuhl für Unternehmenslogistik, Technische Universität Dortmund, Leonhard-Euler-Straße 5, 44227 Dortmund, Deutschland

**Keywords:** Stress, Psychophysiologie, Pausenmanagement, Sensortechnik, Maschinelles Lernen, Stress, Psychophysiology, Break management, Sensor technology, Machine learning

## Abstract

Stress und seine komplexen Wirkungen werden bereits seit Anfang des 20. Jahrhunderts erforscht. Die vielfältigen psychischen und physischen Stressoren in der Arbeitswelt können in Summe zu Störungen des Organismus und zu Erkrankungen führen. Da die Ausprägung körperlicher und subjektiver Folgen von Stress individuell unterschiedlich ist, lassen sich keine absoluten Grenzwerte ermitteln. Zur Erforschung der systematischen Mustererkennung physiologischer und subjektiver Stressparameter sowie einer Stressvorhersage, werden in dem vorliegenden Beitrag Methoden des maschinellen Lernens (ML) eingesetzt. Als praktischer Anwendungsfall dient die Logistikbranche, in der Belastungsfaktoren häufig in der Tätigkeit und der Arbeitsorganisation begründet liegen. Ein Gestaltungselement bei der Prävention von Stress ist die Arbeitspause. Mit ML-Methoden wird untersucht, inwieweit Stress auf Basis physiologischer und subjektiver Parameter vorhergesagt werden kann, um Pausen individuell zu empfehlen. Im Beitrag wird der Zwischenstand einer Softwarelösung für ein dynamisches Pausenmanagement für die Logistik vorgestellt.

*Praktische Relevanz:* Das Ziel der Softwarelösung „Dynamische Pause“ besteht darin, Stress in Folge mentaler und physischer Belastungsfaktoren in der Logistik präventiv vorzubeugen und die Beschäftigten auf lange Sicht gesund, zufrieden, arbeitsfähig und produktiv zu halten. Infolge individualisierter Erholungspausen als Gestaltungselement, können Unternehmen unterstützt werden, Personalressourcen entsprechend der dynamischen Anforderungen der Logistik flexibler einzusetzen.

## Abstract (en)

Stress and its complex effects have been researched since the beginning of the 20th century. The manifold psychological and physical stressors in the world of work can, in sum, lead to disorders of the organism and to illness. Since the physical and subjective consequences of stress vary individually, no absolute threshold values can be determined. Machine learning (ML) methods are used in this article to research the systematic recognition of patterns of physiological and subjective stress parameters and to predict stress. The logistics sector serves as a practical application case in which stress factors are often rooted in the activity and work organisation. One design element of the prevention of stress is the work break. ML methods are used to investigate the extent to which stress can be predicted on the basis of physiological and subjective parameters in order to recommend breaks individually. The article presents the interim status of a software solution for dynamic break management for logistics.

*Practical Relevance:* The aim of the software solution “Dynamic Break” is to preventively prevent stress resulting from mental and physical stress factors in logistics and to keep employees healthy, satisfied, fit for work and productive in the long term. Individualized rest breaks as a design element can support companies in deploying human resources more flexibly in line with the dynamic requirements of logistics.

## Die Bedeutung von Stress für die Logistik

### Stress und seine Wirkungen

Der Stressbegriff wird aufgrund der verschiedenen Wissenschaftszweige, wie Medizin, Biologie, Psychologie, Sozial- und Ingenieurwissenschaften, die sich diesem Thema gewidmet haben, unterschiedlich definiert und verwendet. In dem vorliegenden Beitrag beschreibt Stress einen Zustand im Menschen, der durch erhöhte psychische und/oder physiologische Aktivierung gekennzeichnet ist und sich in einer Störung des dynamischen Gleichgewichts des Organismus, d. h. körperlich, im Erleben und Verhalten äußert (Richter 2000). Stress als natürliche, unspezifische Reaktion des Organismus resultiert daraus, wenn von außen einwirkende Belastungen aller Art im Vergleich zu den verfügbaren Ressourcen von der betroffenen Person negativ interpretiert werden (DIN EN ISO 10075-1:^2018^-01).

Im Arbeitskontext ist unter Belastung die Gesamtheit aller erfassbaren Einflüsse, die von außen auf den Menschen zukommen und psychisch auf ihn einwirken, zu verstehen (DIN EN ISO 10075-1:^2018^-01). Die Belastung einer Arbeit ist zunächst für jeden Menschen wertneutral und beinhaltet verschiedene Anforderungen, die sich aus Einflüssen von Arbeitsbedingungen ergeben, u. a. der Arbeitsaufgabe (z. B. qualitative und quantitative Anforderungen, Zeit- und Termindruck), der Arbeitsorganisation (z. B. günstige Pausengestaltung, unterschiedlicher Arbeitsanfall) oder der Arbeitsplatzgestaltung (z. B. Greifraum, Lastenhandhabung) (Joiko et al. 2010).

Jede Belastung im Individuum führt unmittelbar in Abhängigkeit seiner jeweiligen überdauernden und augenblicklichen Voraussetzungen und individuellen Bewältigungsstrategien (sog. Ressourcen) zu einer Beanspruchung (DIN EN ISO 10075-1:^2018^-01). Beanspruchungen können kurz- und langfristig fördernde (positiv bewertete) und beeinträchtigende (negativ bewertete) Folgen nach sich ziehen. In diesem Rahmen werden außerdem die kurz- oder langfristige Exposition gegenüber der Belastung, die zeitliche Verzögerung zwischen Exposition und Wirkung und die Dauer der Wirkung berücksichtigt (DIN EN ISO 10075-1:^2018^-01).

Unter kurzfristigen positiven Beanspruchungsfolgen ist eine Anregung des Menschen in Form von Aktivierung oder Aufwärmung zu verstehen (Joiko et al. 2010). Liegt ein Ungleichgewicht zwischen hohen Anforderungen einerseits und geringen Ressourcen andererseits vor, entstehen Stressreaktionen und weitere beeinträchtigende Beanspruchungsfolgen, wie z. B. physische und/oder psychische Ermüdung (BAuA 2020). Bei der Entstehung von Stress spielen subjektive Bewertungsprozesse und Prozesse der Bewältigung der Situation eine zentrale Bedeutung, d. h. Stress basiert nicht auf einfachen Ursache-Wirkungs-Zusammenhängen, sondern entsteht intra- und interindividuell unterschiedlich (Richter 2000).

Hinsichtlich kurzfristiger Beanspruchungsreaktionen macht sich Stress auf körperlicher (z. B. Beschleunigung des Herzschlags und der Atmung), emotionaler (z. B. Frustration, Angst, Ermüdungsgefühle) und verhaltensbezogener Ebene (z. B. Konzentrationsstörungen, Fehlerzunahme) bemerkbar. Treten Stressreaktionen häufig und intensiv auf, kann Stress auch längerfristig beeinträchtigende Folgen haben und zur Entwicklung von Erkrankungen oder zum Auftreten von gesundheitlichen Beschwerden, wie muskuloskelettale oder psychovegetative Beschwerden, beitragen (BAuA 2020). Negative Beanspruchungsfolgen im Zusammenhang mit arbeitsbedingtem Stress können sich langfristig in einer erhöhten Anzahl der Unfälle, Krankmeldungen und Fluktuation äußern (Joiko et al. 2010).

### Stressfaktoren in der Logistik und die Wirkung von Arbeitspausen auf die Gesundheit

In einer Auswertung von Certa und Schröder (2020) wird deutlich, dass Beschäftigte in der Lagerwirtschaft häufiger mit Stressoren, die sich aus den physischen und psychischen Arbeitsanforderungen ergeben, konfrontiert sind als Beschäftigte in anderen Berufsgruppen: Zu den physischen Stressoren gehören, im Stehen zu arbeiten, mit schweren Lasten zu arbeiten, in kniender Stellung oder über Kopf zu arbeiten. Bei den psychischen Anforderungen zeigt sich, dass Termin- und Leistungsdruck, Multitasking und ein hohes Arbeitstempo in der Lagerwirtschaft häufiger auftreten als in anderen Berufsgruppen (Certa und Schröder 2020). Der starke Termin- und Leistungsdruck kann dabei eindeutig als Stressor identifiziert werden. Hinsichtlich der Arbeitsfähigkeit, der als abhängige Variable im Rahmen menschengerechter Arbeitsgestaltung eine besondere Relevanz zukommt, wird ersichtlich, dass Beschäftigte in der Lagerwirtschaft eine geringere Arbeitsfähigkeit berichten, die u. a. durch die o. g. Stressoren beeinflusst wird (Certa und Schröder 2020).

Es wird davon ausgegangen, dass Arbeitsbedingungsfaktoren in der Regel in Kombination auftreten und in Wechselwirkung stehen (BAuA 2020). Hinsichtlich der gesundheitsförderlichen Gestaltung von Arbeitssystemen wird empfohlen, primär an sog. Schlüsselfaktoren anzusetzen, die eine starke Wirkung auf andere Arbeitsbedingungsfaktoren haben, wie z. B. der Arbeitszeit (BAuA 2020). Arbeitszeitaspekte können selbst eine Anforderung darstellen, aus der Stress und Stressfolgen resultieren, indem z. B. sowohl die Möglichkeiten als auch die Qualität der Erholung durch die Lage und Länge der Arbeitszeit verringert werden können. Daneben geht mit der Dauer der Arbeitszeit auch die Dauer einher, denen Beschäftigte Arbeitsanforderungen ausgesetzt sind. Die Effekte der Arbeitszeit hängen darüber hinaus mit Einflussmöglichkeiten auf Arbeitszeitmerkmale zusammen. Die Pause repräsentiert vor diesem Hintergrund eine arbeitsbezogene Ressource im Themenfeld „Arbeitszeit“, die zu einer günstigeren Ausprägung von psychischen Gesundheitsindikatoren führen kann (BAuA 2020).

Die Effekte von Arbeitspausen werden bereits seit mehreren Jahrzehnten beforscht. Pausen während der Arbeit dienen dazu, sich von den Folgen der vorausgegangenen Arbeitsbelastung zu erholen. Ein Übersichtsbeitrag von Wendsche und Lohmann-Haislah (2016) verdeutlicht, dass zusätzliche Pausen mit einer Länge von weniger als 15 min, auch als Kurzpausen bezeichnet, muskuloskelettale Beschwerden bzw. Erkrankungen und stressbezogene kardiovaskuläre Indikatoren reduzieren oder vorbeugend verhindern können. Die Konzentrations‑, Aufmerksamkeits- und Gedächtnisleistung profitiert ebenfalls von kurzen Pausen. Damit einhergehend konnten vereinzelte präventive und beanspruchungsoptimierende Effekte von Kurzpausenregimen aufgezeigt werden, die sich z. B. in Form einer niedrigeren kardiovaskulären Aktivierung äußern.

Daneben wird deutlich, dass sich arbeitszeitliche Ressourcen, wie z. B. Einfluss nehmen auf die Arbeitszeit, positiv auf die subjektive Erholung und die Gesundheit auswirken können (BAuA 2020). Dennoch weisen Studien darauf hin, dass selbstorganisierte kurze Pausen häufig zu spät, d. h. erst bei erlebten körperlichen Beschwerden und einem deutlichen Erschöpfungserleben, genommen werden (Wendsche & Lohmann-Haislah 2016). Empfehlungen für zukünftige Untersuchungen zu Arbeitspausen gehen dahin, neben Selbstauskünften der Beschäftigten auch physiologische Messmethoden miteinzubeziehen. Vor dem Hintergrund zum Forschungsstand zu Arbeitspausen kann konstatiert werden, dass sich Arbeitspausen positiv auf die Gesundheit, das Befinden, die Motivation und die Leistung von Beschäftigten auswirken sowie das Risiko von Arbeitsunfällen, Fehlzeiten oder Mitarbeiterfluktuation abnimmt (Wendsche & Lohmann-Haislah 2016). Entsprechend der Befundlage zu gesundheitlichen Effekten von Arbeitspausen, können kurze Erholungspausen als geeignete Interventionsmaßnahme im Rahmen der betrieblichen Prävention und einer gesundheitsfördernden Organisationsentwicklung gesehen werden (Faller 2010). Im Falle selbstorganisierter Erholungspausen kann die Arbeitspause zudem als ein Element der betrieblichen Gesundheitsförderung eingesetzt werden (Faller 2010).

## Motivation und Projektvorstellung „Dynamische Pause“

### Potenziale des Einsatzes von KI und Wearable-Sensoren für die Vorhersage von Stress

Künstliche Intelligenz (KI) ist ein Mantelbegriff, welcher Anwendungen zusammenfasst, die ein intelligentes Verhalten zeigen. Die Potenziale und Anwendungsmöglichkeiten für KI werden aktuell umfangreich erforscht. Neben Chancen für neue Geschäftsmodelle mit disruptivem Charakter, bietet KI außerdem Optimierungspotenziale zur Steigerung von Effizienz und Automatisierung von Wertschöpfungsketten (Bitkom 2018). Dennoch ist ein konkreter Einsatz von KI-Anwendungen laut einer Studie des Fraunhofer-Instituts für Arbeitswirtschaft und Organisation bislang nur bei jedem sechsten Unternehmen der Fall (Bauer et al. 2019). Der Mehrwert und die Potenziale von KI für eine gesunde, produktive und sichere Arbeitsgestaltung wurde in internationalen Forschungsarbeiten bereits bestätigt, wodurch das Risiko für Unternehmen ohne KI steigt, im Vergleich dazu, den Anschluss zu verlieren (Frost et al. 2020).

Als Teilbereich der KI ist das maschinelle Lernen (auch: Machine Learning, ML) in der Lage, basierend auf Daten, Klassifizierungen vorzunehmen, Prognosen zu errechnen und Zusammenhänge zu erlernen. Mittels einer bestimmten Kategorie von Algorithmen werden Statistiken verwendet, um Muster in großen Datenmengen zu finden. Auf der Basis von großen, unstrukturierten Datenmengen werden Regelmäßigkeiten, Wiederholungen oder Ähnlichkeiten in Daten maschinell erfasst und daraus relevante Informationen gewonnen. Zur Lösung einer Lernaufgabe muss aus einer Vielzahl an potenziellen Lernverfahren (Algorithmen), ein passender Algorithmus angewendet werden (Döbel et al. 2018).

In der Praxis verläuft der Weg in die Anwendung über die Erhebung und Bereinigung von Daten, die Identifikation passender Algorithmen, sowie der Evaluation der Qualität des berechneten Modells. Mit dem Ziel der Klassifizierung, erlernt ein statistisches Modell aus vorhandenen Daten Zusammenhänge und Gesetzmäßigkeiten und kann diese auf neue, nicht vorher gesehene Daten anwenden. Das Ergebnis ist ein statistisches Modell, welches unbekannte Daten selbständig klassifizieren kann. In diesem Rahmen wird auch von der Generalisierbarkeit des Modells gesprochen (Wennker 2020). Das Kernproblem ist somit, ein Modell zu finden, welches aus bekannten Daten die notwendigen Zusammenhänge erlernt und auf neuen, unbekannten Daten hinreichend genau generalisiert.

Die Potenziale von ML für verschiedene medizinische Anwendungsfälle wurden in Studienergebnissen bereits veröffentlicht. So ist es möglich, durch ML physiologische Daten in Echtzeit zu verarbeiten (Attaran et al. 2018), Migräne frühzeitig zu erkennen (Koskimäki et al. 2017), sowie das Stresslevel von Patienten vor Operationen im Vorfeld zu bestimmen (Anusha et al. 2019). Eine große Rolle in diesem Kontext spielt der Einsatz von Wearable-Sensoren als Datenquelle für die ML-Verarbeitung. Insbesondere im Bereich der Epilepsie- und Alzheimerforschung wurden zahlreiche Untersuchungen unter Einsatz von Wearable-Sensoren durchgeführt. So konnten durch den Einsatz von Bewegungssensoren an den Füßen, Beinen und an der Hüfte über eine Gang- und Balanceuntersuchung Merkmale einer Alzheimererkrankung erkannt werden (Hsu et al. 2014; Margiotta et al. 2016). Kourtis et al. (2019) weitet diese Betrachtung aus und untersuchte, welche Biomarker sich für die frühzeitige Erkennung einer Alzheimererkrankung heranziehen lassen. Neben der Bewegungsanalyse wurden Wearable-Sensoren verwendet, um Schäden im zentralen Nervensystem über Vitalwerte, wie die Herzratenvariabilität (HRV), identifizieren zu können. Im Bereich der Erkennung und Dokumentation epileptischer Anfälle wird ebenfalls Sensortechnik am Körper eingesetzt. Im Fokus dieser Entwicklungen sind technische Lösungen, die im Alltag verwendet werden können, und so eine Alternative zu dem sonst üblichen Klinikaufenthalt des Patienten ermöglichen. Die Arbeit von Rukasha et al. (2020) erläutert, dass die Kombination aus Herzrate (HR), elektrodermaler Aktivität (EDA) sowie Bewegungen zuverlässige Rückschlüsse für die Erkennung epileptischer Anfälle ohne den Einsatz eines EEG ermöglicht. So wirken sich Anfälle neben den typischen Bewegungsmustern insbesondere auch auf die EDA aus.

Nach aktuellem Stand der Forschung wurden Stress und sein Entstehungs- und Wirkungsprozess nicht vollumfänglich zum Training von ML-Modellen einbezogen. Um diese Lücke zu schließen, wurde im Folgenden zusätzlich eine Selbstauskunft der Probanden während der Studie erhoben, um die Klassifizierung von Stress als negative Beanspruchungsfolge und Aktivierung als positive Beanspruchungsfolge zu gewährleisten. Diese erweiterte Datengrundlage ist die Basis für diesen Fachbeitrag, in dem ein generalisierendes ML-Modell zur Detektion von Stress und Prädiktion von kritischen Stresslimits erstellt wurde.

### Das Entwicklungsprojekt „Dynamische Pause“

Die „Dynamische Pause“ fügt sich in die Entwicklungsprojekte der „Silicon Economy Logistics Ecosystem“ ein. Das vom BMVI geförderte Großprojekt hat sich der Entwicklung von hochwertigen Open Source-Anwendungen im Bereich von Logistik und Supply Management verschrieben. In der sehr technologieorientierten und heterogen geprägten Logistikbranche spielt die Erholungspause im Arbeitskontext eine wichtige Rolle. Flexibilität ist eine Notwendigkeit, um den zeitkritischen Betrieb aufrecht zu erhalten und gleichzeitig wirksam auf unvorhergesehene Veränderungen zu reagieren. Beispiele dafür finden sich in Kommissionierungsprozessen, in welchen die gleichverteilte Auslastung von menschlichen Akteuren und maschinell gesteuerten Verteilsystemen effizient gestaltet werden muss. Die „Dynamische Pause“ hat die Aufgabe den unerwünschten Effekten von Stress und nicht eingehaltenen Pausen entgegenzuwirken und gleichzeitig die Effizienz der Prozesse zu erhalten. Auf Basis der in Echtzeit ausgewerteten Vitaldaten werden den Beschäftigten individuelle Pausen empfohlen. Diese werden über ein Wearable gemessen und mit ML-Methoden ausgewertet. Ziel ist es, den persönlichen Stressanstieg vorauszusagen, damit rechtzeitig eine Pause vorgeschlagen werden kann. Die Empfehlung erfolgt über eine proprietäre Smartphone-App, welche die Interaktion mit dem System ermöglicht. Die Pausen werden durch einen Ressourcen Management-Service (RMS) orchestriert, welcher die Daten in einer Cloud auswertet und dabei die anderen Beteiligten des Arbeitssystems im Blick behält. Die Auswertung der Daten erfolgt anonym, ein Datenschutzkonzept gemäß Datenschutz-Grundverordnung (DSGVO) sorgt für die für die Einhaltung der gesetzlichen Vorgaben zum Datenschutz und der Datensicherheit beim Umgang mit personenbezogenen Daten. Ein weiteres Ziel des Projektes der „Dynamischen Pause“ ist es, die Arbeitsorganisation in Unternehmen gleichermaßen an die unternehmensinternen Prozesse und Anforderungen der Logistik sowie an die Bedürfnisse der Beschäftigten anzupassen. Es ist zukünftig angedacht, die „Dynamische Pause“ in die bereits existierende IT-Umgebung zur Steuerung und Planung der innerbetrieblichen Materialflusssysteme zu integrieren. Der Dienst der „Dynamischen Pause“ bietet das Potenzial, den RMS mit betriebsinternen Tools zur Ressourcenplanung und -steuerung zu verknüpfen, sodass langfristig die Gesamtheit an Ressourcen, d. h. sowohl Aufträge als auch Mitarbeitende, aufeinander abgestimmt eingesetzt werden können.

Im Folgenden wird der Zwischenstand des laufenden Entwicklungsprojektes präsentiert. Der Beitrag beschreibt den aktuellen Forschungsstand im Projekt bezüglich der Auswahl von Methoden zur Messung physiologischer und subjektiver Stressdaten sowie der Auswahl geeigneter ML-Methoden für die Analyse der Stressparameter. Das Phänomen „Stress“ und seine komplexen Wirkungen wird bereits seit Anfang des 20. Jahrhunderts erforscht. Eine Herausforderung bei der Analyse von Stress ist bis heute, dass die Ausprägung körperlicher und subjektiver Folgen von negativem Stress individuell unterschiedlich ist und sich keine absoluten Grenzwerte ermitteln lassen. Das Projekt wagt einen explorativen Vorstoß in die Erforschung von Stress durch die Nutzung von KI-Methoden zur Analyse objektiver und subjektiver Daten, um Stress zu messen, zu detektieren und kritische Stresslimits vorherzusagen.

## Studie zur Gewinnung von physischen und psychischen Stressdaten

Für die Gewinnung von physischen und psychischen Stressdaten wurde eine Laborstudie durchgeführt. Die Operationalisierung von Stress erfolgt mittels objektiver und subjektiver Daten. Im Folgenden wird ein Überblick über Messmethoden von Stress gegeben sowie die durchgeführte Untersuchung beschrieben.

### Übersicht der Messmethoden von Stress

Zur Quantifizierung von Stress kann zwischen subjektiven und objektiven Datenerhebungsaspekten unterschieden werden. Kurzfristige Beanspruchungsreaktionen des Menschen auf körperlicher Ebene lassen sich mit objektiven, physiologischen Daten operationalisieren. Zu den zentralen psychophysiologischen Reaktionssystemen des Menschen zählen elektrodermale, kardiovaskuläre Muskel- und hirnelektrische Aktivitäten (Fahr und Hofer 2013). Es werden Messverfahren eingesetzt, die physiologische Reaktionen des Menschen objektiv, z. B. durch Messelektroden oder -dioden auf verschiedenen Ableitorten am Körper, in einer Belastungssituation erfassen. Im Folgenden konzentrieren wir uns auf elektrodermale und kardiovaskuläre Aktivitäten, da diese am häufigsten in der Wirkungsforschung eingesetzt werden (Fahr und Hofer 2013).

Die Elektrodermale Aktivität (EDA) ist mit zentralen psychologischen Phänomenen, wie Aktivierung, Aufmerksamkeit, Informationsverarbeitung und emotionale Reaktionen, assoziiert (Fahr und Hofer 2013). Zu den wichtigsten EDA-Kennwerten in der Wirkungsforschung gehören das Niveau der Hautleitfähigkeit und seine Änderungen (SCL = Skin Conductance Level) sowie die sog. Skin Conductance Responses (SCR). Bei der Messung des Potenzials der Haut selbst kommen traditionelle und moderne Technologien, wie Sensorarmbänder, zum Einsatz.

Neben der EDA wird auch das Reaktionssystem der kardiovaskulären Aktivität (KVA) verwendet (Fahr und Hofer 2013). Ähnlich wie bei der EDA wird eine Korrelation zwischen physischen Merkmalen und psychischen Prozessen des Menschen, wie Aktivierung, Aufmerksamkeit, Habituation, Stress oder Emotionen, berichtet (Fahr und Hofer 2013). Zu den wichtigen Parametern der KVA zählen u. a. die Herzschlagfrequenz (engl. heart rate (HR), angegeben in Schlägen pro Minute, engl. beats per minute (bpm)), die periphere Durchblutung bzw. das periphere Blutvolumen (PBV) sowie der Blutdruck. Neben der traditionellen Messung des Elektrokardiogramms (EKG), z. B. an der Brustwand, kann die KVA mit photoplethysmographischen (PPG) Messungen an z. B. Ohrläppchen oder Fingerkuppen erhoben werden. Empfohlene Kennwerte sind der Inter-Beat-Intervall (IBI), der Abstand zwischen den zwei sog. R‑Zacken (RR-Abstand), oder die aus HR oder IBI abgeleitete Herzratenvariabilität (HRV) (Fahr und Hofer 2013).

Da sich Stress auch auf emotionaler Ebene bemerkbar macht, z. B. in Form von Frustration, Angst oder Ermüdung, werden auch zusätzlich subjektive Daten herangezogen. Hier kommen Verfahren zum Einsatz (z. B. Fragebögen oder Interviews), die die Daten des subjektiven Erlebens einer Belastungssituation eines Menschen erfassen. Die Aktiviertheit des Menschen stellt ein vielschichtiges Konstrukt dar, wofür es sowohl auf psychophysiologischer als auch auf psychologischer Betrachtungsebene keine eindeutigen Kriterien gibt. Eine Herausforderung besteht zudem darin, dass objektiv ähnliche psychophysiologische Zustände auf intra- und interindividueller Ebene unterschiedlich subjektiv wahrgenommen werden.

### Auswahl der Sensortechnik für die objektive Stressmessung

Die Forschung beschäftigt sich bereits seit mehreren Jahren mit der Stresserkennung anhand von Vitaldaten. Ziel ist die Identifizierung von Stressphasen eines Menschen. Bereits 2010 wurde durch Shi et al. (2010) die Erkennung von Stress durch die Erhebung physiologischer Daten untersucht (EKG, EDA, Atmung, Hauttemperatur). In einer Studie wurden objektive und subjektive Daten erhoben, um durch den Einsatz maschinellen Lernens Stress identifizieren zu können. Sierra et al. (2011) verwendet ebenfalls Vitaldaten für die Stresserkennung und setzt hierfür ein Sensorarmband zur Messung der HR und EDA ein. Durch die gezielte Stressorinduktion, wie Hyperventilation und emotionale Gespräche, wurden in einer Studie Stressphasen geschaffen, die durch den Einsatz von Fragebögen und der Messung der Vitaldaten dokumentiert werden konnten. Mit Hilfe maschinellen Lernens konnte eine hohe Erkennungsrate von bis zu 99 % für die Identifikation von Stressphasen erreicht werden (Sierra et al. 2011).

Erreichen Methoden zur Stresserkennung in eingeschränkten Umgebungen eine hohe Genauigkeit, sinkt diese, sobald die Stresserkennung in unbeschränkten Umgebungen – also dem realen Leben – erfolgen soll. So beschreibt Can et al. (2019), dass die Genauigkeit der Algorithmen im realen Leben deutlich abnimmt und sich nur noch zwischen 70 und 80 % bewegt. Gjoreski et al. (2017) gibt an, dass sich die Differenzierung einzelner Stressphasen in unbeschränkten Umgebungen deutlich schwieriger gestaltet. Sowohl Can et al. (2019) als auch Gjoreski et al. (2017) stellen in ihren Arbeiten Lösungsansätze vor, die eine zuverlässige Stresserkennung im realen Leben durch den Einsatz von Wearable-Sensoren, ermöglichen. Die Autoren haben in ihren Forschungen ein Sensorarmband verwendet, um den Blutvolumenpuls (BVP), die EDA, die Hauttemperatur sowie die Beschleunigung zu erfassen. Für das Training von ML wurde darüber hinaus eine App verwendet, mit der vier bis sechs Mal am Tag, in zufälligen Abständen, der aktuelle Stresswert durch subjektive Einschätzung des Probanden angegeben werden musste. Für die Stresserkennung wurden außerdem verschiedene Parameter berücksichtig, um den jeweiligen Kontext des Probanden zu identifizieren. Dazu gehörte z. B. wie lange die letzte körperliche Aktivität zurücklag und in welcher Tageszeit sich der Proband befunden hat. Mit diesem Ansatz erreichte Gjoreski et al. (2017) eine Genauigkeit von 95 %. Auch Can et al. (2019) hat Wearable-Sensoren verwendet, um die Stresserkennung in Alltagssituationen zu realisieren und verwendete Kontextdaten, um die gemessenen Daten zu interpretieren. Jedoch beschränkte sich dieser Ansatz auf einen fest definierten Tagesablauf, der aus Vorlesungen und einer Prüfungssituation bestand. In diesem Rahmen wurden die Vitaldaten der Probanden erfasst (BVP, EDA und Beschleunigung). Außerdem wurde durch die Probanden nach jeder Vorlesung sowie nach der Prüfung ein Fragebogen zur Belastungsmessung ausgefüllt. Mit diesem Vorgehen und der Anwendung eines Algorithmus des maschinellen Lernens (hier: Multilayer Perceptron) konnte eine Genauigkeit von knapp über 92 % erreicht werden.

In einem Review zum Thema Stresserkennung anhand von Vitaldaten wurde durch Can et al. (2019) analysiert, welche Methoden des maschinellen Lernens für das Training des Erkennungssystems herangezogen wurden und welche Vitalparameter zum Einsatz kamen. Von 13 Veröffentlichungen, die sich mit der Stresserkennung im realen Leben beschäftigt haben, verwendeten neun einen EDA- und fünf einen PPG-Sensor. Insbesondere die Werte des PPG-Sensors wurden für die Ermittlung verschiedener Feature herangezogen. Zu diesen Features gehörten die HR, die HRV sowie der BVP. Für das Training der Erkennungssysteme kamen insbesondere Support Vector Machines (SVM), K‑Nearest-Neighbor (KNN) sowie Bayes-Netze zum Einsatz. Es konnte jedoch auch der Einsatz von Entscheidungsbäumen, künstlichen neuronalen Netzen sowie Random Forest beobachtet werden.

Sowohl im medizinischen Kontext als auch bei der Forschung zur Stresserkennung wird auf Basis bestehender Entwicklungen und Studien deutlich, dass die Auswahl geeigneter Wearable-Sensortechnik begrenzt ist. Am häufigsten zum Einsatz kam in den betrachteten Studien die Empatica E4 (z. B. Gjoreski et al. 2017), ein Armband das explizit für die Forschung entwickelt wurde und das als eines der wenigen Endgeräte die Messung des EDA am Handgelenk für den mobilen Einsatz ermöglicht. Neben dem EDA-Sensor verfügt das Armband über einen PPG-Sensor, mit dem sich verschiedene Features erfassen lassen (u. a. BVP, HR, HRV). Ein Temperatursensor ermöglicht die Messung der Hauttemperatur und ein integriertes Gyroskop erfasst die Beschleunigung in x‑, y‑ und z‑Richtung. Hiermit unterstützt das Armband die Messung aller Vital- und Aktivitätsdaten, die in bestehenden Entwicklungen zur Stressmessung in der Realität zur Verwendung kommen. Hierzu gehören insbesondere Kennwerte, wie HR, BVP, EDA, Hauttemperatur und Bewegungen. Auf Basis der betrachteten Erkenntnisse wurde für die durchgeführte Studie der „Dynamischen Pause“ die Empatica E4 als Sensorarmband für die Erfassung von Vitaldaten im Kontext der Stresserkennung ausgewählt, das eine hohe Messgenauigkeit aufweist (Menghini et al. 2019).

### Auswahl der Fragebögen für die subjektive Stressmessung

Da Stressreaktionen intra- und interindividuell unterschiedlich entstehen und sich äußern können (vgl. Abschn. 1.1), wurden Fragebögen zur Operationalisierung des subjektiven Empfindens von Stress ausgewählt, die das Konstrukt aus verschiedenen Perspektiven betrachten. Die subjektiven Daten dienen dazu, die Vorhersage von Stress zu verbessern, indem der Erkenntnisgewinn bzgl. der psychophysiologischen Aktivierung erhöht wird.

Bei der Klassifizierung von Stress ist es wichtig, die Art und den Umfang der zu Grunde liegenden Stressoren, die mit hoher Wahrscheinlichkeit zu Stress führen, zu erkennen (vgl. Abschn. 1.1). Von Interesse war außerdem, ob sich das subjektive Stressempfinden bei psychischen Stressoren von dem bei physischen Stressoren unterscheidet oder ein gleiches Muster nach sich zieht. Zur Bewertung der geistigen und körperlichen Belastungen sowie der Anstrengung und Frustration wurde der NASA-Task Load Index verwendet (Staveland und Hart 1988). Da bekannt ist, dass die Wachheit unter Stress erhöht wird, wurde das Ausmaß der Aktivierung der Probanden mit einer Skala zur Tagesschläfrigkeit überprüft (Stanford Sleepiness Scale, Hoddes et al. 1973).

Ein weiteres Ziel war es, Stress als beeinträchtigende Beanspruchungsfolge von Aktivierung als positive Beanspruchungsfolge zu unterscheiden. Für die Messung der Affektivität wurde die Positive And Negative Affect Schedule (Krohne et al. 1996) eingesetzt, welche momentane positive und negative Empfindungen und Gefühle bewertet. Untersuchungen liefern Hinweise darüber, dass Zusammenhänge zwischen sowohl positivem und negativem Affekt und physiologischem Stress und Gesundheitsoutcomes (z. B. koronare Herzerkrankungen) anzunehmen sind (Brouwers et al. 2013).

Des Weiteren kam die Skala „Stress“ der Depression Anxiety and Stress Scale (Nilges und Essau 2015) zum Einsatz. Diese misst ein spezifisches Syndrom, das sich faktoriell von Depression und Angst unterscheidet und durch nervöse Anspannung, Entspannungsschwierigkeiten und Reizbarkeit gekennzeichnet ist. In Studien konnten Zusammenhänge zwischen Stress und der psychischen Gesundheit, Vitalität und sozialer Funktionsfähigkeit nachgewiesen werden (Ng et al. 2007).

### Untersuchungsdesign und Durchführung einer Studie zur Induktion von Stress

**Figure 1:**
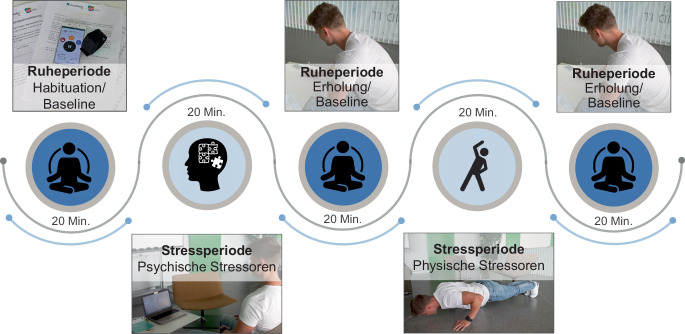

Zur Erhebung und Bewertung individueller Stressreaktionen auf körperlicher und emotionaler Ebene bei physischer und psychischer Belastungsexposition wurde vor dem Hintergrund der COVID-19 Situation zunächst eine Studie in einem laborexperimentellen Kontext durchgeführt (Abb. 1). Diese basiert auf Erkenntnissen experimenteller Stressforschung.

Ziel der Studie war es, physiologische und emotionale Reaktivität zu induzieren, d. h. die individuellen körperlichen und emotionalen Reaktionen auf physische und psychische Stressreize zu erkennen und hinsichtlich ihres Ausmaßes zu unterscheiden. Studienergebnisse experimenteller Stressforschung zeigen, dass verschiedene Stressoren eingesetzt werden, um Stressreaktionen zu induzieren (Giannakakis et al. 2019). Vor dem Hintergrund der häufig auftretenden psychischen und physischen Arbeitsbelastungsfaktoren in der Logistik, wurden Stressoren ausgewählt, die sich auf die Arbeitsaufgaben beziehen, und verschiedene Stressbedingungen dargeboten.

**Figure 2:**
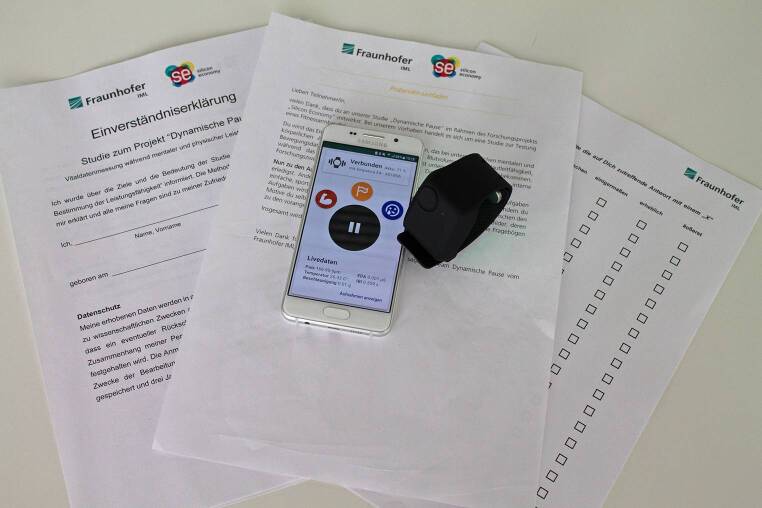

Um mögliche suggestive Effekte zu vermeiden, wurde die Studie als „Vitaldatenstudie“ bezeichnet. Nach der ausführlichen Aufklärung zum Studienablauf gaben die Probanden ihr schriftliches Einverständnis zur Teilnahme an der Studie, unterzeichneten die Datenschutzerklärung und wurden gebeten, das Empatica-Sensorarmband an dem nicht dominanten Handgelenk anzulegen (Abb. 2).

Die Studie begann mit einer 20-minütigen Ruheperiode, die die Funktion der Habituation (10 min) und der Gewinnung der Baseline der subjektiven und objektiven Daten hatte (10 min). In Anlehnung an bisherige Forschung (Zbozinek et al. 2015) wurden zur Entspannung positiv valente Stimuli in Form von Bildern dargeboten, die vorher persönlich abgefragt wurden (z. B. Berge, Meer, Sonnenuntergang). Weitere Aktivitäten der Ruhephase konnten frei gewählt werden (z. B. Lesen). Am Ende der Ruheperiode beantworteten die Probanden Fragebögen zu subjektiven Konzepten (vgl. Abschn. 3.2).

Im Anschluss an die erste Ruheperiode folgte eine 20-minütige Stressinduktionsphase, die aus verschiedenen Kombinationen von Stressoren bestand. Die Reihenfolge physischer sowie psychischer Stressoren wurde randomisiert, um additive Effekte auf Stressreaktionen auszubalancieren (Finke et al. 2021). Nach jeder Stressphase folgte eine erneute 20-minütige Ruheperiode, in der die Probanden aufgefordert wurden, die Belastung der mentalen sowie körperlichen Aufgaben sowie ihren subjektiven Zustand mit Fragebögen zu bewerten, während sie sich erholten (10 min). In der zweiten Hälfte der Ruheperiode wurden die Probanden zur Messung der Baseline der Vitalparameter erneut instruiert, sich zu entspannen (10 min). In der Stressperiode „Psychische Stressoren“ wurden zwei standardisierte Verfahren mit kognitiven Stressoren und ein individualisiertes Verfahren mit einem emotionalen Stressor eingesetzt. Standardisierte kognitive Reize werden vorwiegend mittels arithmetischer Aufgaben oder Aufmerksamkeits- und Konzentrationsaufgaben induziert, da sie mit einer Beanspruchung kognitiver Ressourcen einhergehen. Der erste kognitive Stresstest basierte auf der arithmetischen Aufgabe, in 7er Schritten ab 100 rückwärts zu zählen (Finke et al. 2021). Als Aufmerksamkeitsaufgabe wurde der bereits vielfältig eingesetzte Farbe-Wort-Interferenztest (Stroop-Test) in Form einer validierte App-basierten Variante „EncephalApp“ als Aufgabe gestellt (Bajaj et al. 2015). Bei beiden kognitiven Stresstests wurden soziale Stressoren, wie soziale Evaluation (Erfolgskontrolle und Zeitmessung) und Motivation durch die Versuchsleitung sowie Unkontrollierbarkeit der Aufgabe als Moderator eingesetzt, da diese das Ausmaß physiologischer Reaktivität zusätzlich intensivieren können. Allein die Anwesenheit einer Person, das Risiko einer negativen Bewertung oder eine gefühlte fehlende Kontrolle trotz starker Bemühungen können Stressreaktionen verstärken (Hellhammer et al. 2010). Bei der dritten Stressaufgabe wurde eine Kombination aus einem individualisierten emotionalen und einem sozialen Reiz gewählt. Die Probanden hatten die Aufgabe, der Versuchsleitung persönliche Themen, wie z. B. negative Lebensereignisse oder interpersonelle Konflikte aus dem privaten oder beruflichen Bereich zu erzählen (Blackhart et al. 2007). In der Stressperiode „Physische Stressoren“ hatten die Probanden die Aufgaben Treppen zu steigen (Yuen et al. 2021) sowie vorgegebene Fitnessübungen auf Zeit zu bewältigen (Giannakakis et al. 2019).

## Ergebnisse der ML-basierten Analysen

Nachfolgend werden die ersten Ergebnisse der durchgeführten Studie zur Gewinnung von subjektiven und objektiven Stressdaten vorgestellt. Im folgenden Beitrag wird vordergründig auf die Ergebnisse der ML-basierten Analysen der Vitalparameter eingegangen. Hierbei wird zunächst die Zeitreihen-Klassifikation vorgestellt. Die Überprüfung anderer ML-Ansätze stehen zukünftig noch aus. Die Integration der subjektiven Daten in das verwendete ML-Modell zur Verbesserung der Detektion bzw. Vorhersage von Stress steht in einem weiteren Schritt aus, der in diesem Beitrag nicht thematisiert wird.

### Datenbestand

Die Stichprobe bestand aus *N* = 32 Probanden, wobei die Anzahl teilnehmender Männer und Frauen ausgeglichen war. Die Mehrheit der Probanden befand sich in der Altersgruppe der 25- bis 34-Jährigen (62,5 %), jeweils 18,75 % waren 24 Jahre und jünger sowie 35 Jahre und älter (30,8 ± 10,5 Jahre). Die Daten des PPG-Sensors wurden softwareseitig mit Hilfe eines Bandpasses gefiltert. Nach Kiselev et al. (2020) wurde dabei das Frequenzband von 0,04 bis 4 Hz gewählt. Zudem wurden die Zeitreihen auf einen Bereich zwischen 0 und 1 skaliert, um die Vergleichbarkeit zwischen Probanden sowie innerhalb der Zeitreihen zu verbessern.

### Windowing und Labeling der Daten

Zur Detektion und Vorhersage von Stress standen im Projekt „Dynamische Pause“ verschiedene Daten zur Verfügung, welche unterschiedliche Beiträge für den KI-Algorithmus liefern. Durch das Sensorarmband selbst können das BVP-Signal, der EDA-Wert, die Körpertemperatur und die Beschleunigung der Bewegung in x‑, y‑ und z‑Achse erfasst werden. Die Laborstudie liefert darüber hinaus sowohl objektive als auch subjektive Daten. Die objektiven Daten markieren die Zeitfenster, in denen bestimmte Stressoren induziert wurden. Verarbeitet werden die Daten im ML-Modell durch überwachtes Lernen. Hierbei werden die Daten gezielt in Zeitfenster unterteilt, sog. „Windowing“. Die Größe dieser Fenster basiert zunächst auf einem Erfahrungswert, während der Verfeinerung des Models können nachträglich angepasst werden. Die Studie von Meisel et al. (2020) schlägt für die Daten der Empatica Fenstergrößen von 150 bis 1200 s vor und erzielte die besten Ergebnisse bei 600 s. Aus diesen Fenstern lassen sich Samples extrahieren, die dem Algorithmus als Lernbeispiele dienen. Eine größere Schrittgröße führt zu einer geringeren zeitlichen Überlappung der Samples. Bei einer großen Überlappung steigt das Risiko einer Überanpassung innerhalb des Lernprozesses.

### Auswahl eines maschinellen Lernverfahrens

Das Ziel von ML besteht darin, auf Basis einer Datenmenge ein diskriminatives Modell zu ermitteln, welches eine Abbildung von Vitalparametern auf dem entsprechenden Stresslevel realisiert und dabei den Klassifikationsfehler minimiert. Die Parametrisierung eines Modells wird dabei auf Basis der zur Verfügung stehenden Daten trainiert. Dabei kommen je nach Algorithmus und Modell unterschiedliche Optimierungsstrategien und -kriterien zum Einsatz.

Eine Herausforderung für die Klassifikation stellt dabei das Format der Daten dar. Während klassische ML-Ansätze von einem Sample bestehend aus voneinander unabhängigen Komponenten ausgehen, weisen Zeitreihen durch den zeitlichen Verlauf eine starke Abhängigkeit von aufeinander folgenden Werten dar. Um mit Zeitreihendaten umgehen zu können, wurden im Rahmen dieses Beitrags zwei Ansätze untersucht: Direkte Zeitreihen-Klassifikation und Deep Learning. Die Ansätze werden im Folgenden genauer behandelt.

#### Zeitreihen-Klassifikation

Während bei klassischen Modellen und Algorithmen des ML die Merkmale, die als Input des Modells dienen, selbst entworfen werden müssen, setzen Ansätze der Zeitreihen-Klassifikation auf eine direkte Analyse der Daten. Hierbei kommen auf Zeitreihen angepasste Modelle zum Einsatz.

Um mit multivariaten Zeitreihen umgehen zu können, bieten sich zwei Ansätze an: Ensembling und Konkatenation. Bei ersterem wird für jede in einem Sample befindliche Zeitreihe ein separater Klassifikator trainiert, wodurch in dem betrachteten Anwendungsfall vier Klassifikatoren generiert werden. Die Entscheidung eines Ensembles wird dann über Mehrheitsentscheid der Klassifikatoren gefällt. Der Ansatz der Konkatenation verbindet durch Aneinanderreihung vier Zeitreihen zu einer gesamten Zeitreihe. Auf der zusammengesetzten Zeitreihe genügt ein Klassifikator. Hierbei soll zum einen ein Nächste-Nachbar-Klassifikator zum Einsatz kommen, der als Abstandsmaß Dynamic Time Warping verwendet (Xi et al. 2006). Ein weiterer untersuchter Ansatz setzt dabei auf Random Forests, die auf statistischen Merkmalen der Zeitreihen trainiert werden (Deng et al. 2013). Der dritte und letzte Ansatz über Random Interval Spectral Forests nutzt dabei, anders als die ersten beiden Ansätze, den Frequenzbereich der Zeitreihen, auf Basis dessen Random Forests trainiert werden (Lines et al. 2018). Im Rahmen dieses Beitrags werden beide Modelle jeweils mit Ensembling und Konkatenation untersucht. Aus Gründen der Übersichtlichkeit, wurden die jeweiligen Ensembles mit homogenen Klassifikatoren trainiert.

#### Deep Learning Klassifikation

Deep Learning stellt im Bereich der Zeitreihen-Klassifikation einen Sonderfall dar. Modelle dieser Art nehmen rohe Zeitreihen als Input, nutzen aber vor der eigentlichen Klassifikation eine interne Merkmalsextraktion. Diese wird nicht implementiert, sondern zusammen mit dem Klassifikationsmodell trainiert. Durch die gemeinsame Optimierung sind Merkmalsextraktion und die anschließende Klassifikation aufeinander abgestimmt. Insbesondere in Bereichen wie der Bildverarbeitung konnten durch Deep Learning Erfolge erzielt werden (Mahony et al. 2020). Eine Vielzahl verschiedener Architekturen neuronaler Netze lässt sich zur Klassifikation von Zeitreihen einsetzen (Fawaz et al. 2019). Dieser Beitrag fokussiert sich auf die Architektur des Convolutional Neural Network (CNN) nach Wang et al. (2016).

**Figure 3:**
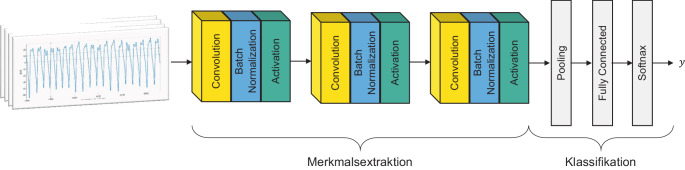

Zur Klassifikation werden aus den Zeitreihen hierbei zunächst über drei sequenziell angeordnete Faltungsblöcke relevante Merkmale extrahiert, ehe diese mittels einer vollvernetzten Schicht einer Klasse zugewiesen werden (Abb. 3).

### Evaluierung

Zu jedem der vorgestellten Algorithmen wurde ein separates Hyperparameter-Tuning durchgeführt, welches die jeweilige Leistung optimieren sollte. Neben der algorithmenspezifischen Hyperparameter wurden zudem die Fenstergröße und Schrittweite des Windowings als Parameter ins Training miteinbezogen. Dadurch sollte das Verhalten der Algorithmen auf unterschiedlichen Datenmengen untersucht werden.

Für jeden Trainingslauf wurde der Datensatz *D* gleichverteilt in zwei Subdatensätze *D*_*Train*_ und *D*_*Test*_ im Verhältnis 80 zu 20 gespalten. Ersterer dient dabei als Grundlage für das eigentliche Training des Modells. Da *D*_*Test*_ nicht im Training beachtet wird, dient dieser zur Evaluierung der Generalisierungsleistung des Modells. Aufbauend auf den jeweiligen Vorhersagen eines Modells auf *D*_*Test*_ lassen sich Metriken bestimmen, anhand derer die Leistung verschiedener Modelle quantifiziert werden kann. Im Fokus steht hierbei der Korrelationskoeffizient nach Matthews (1975). Dieser gibt die Korrelation zwischen der Vorhersage eines Modells und der tatsächlichen Label wieder. Der Vorteil hierbei ist, dass dieser sich robust gegenüber unbalancierten Klassen verhält (Chicco und Jurman 2020).

**Figure 4:**
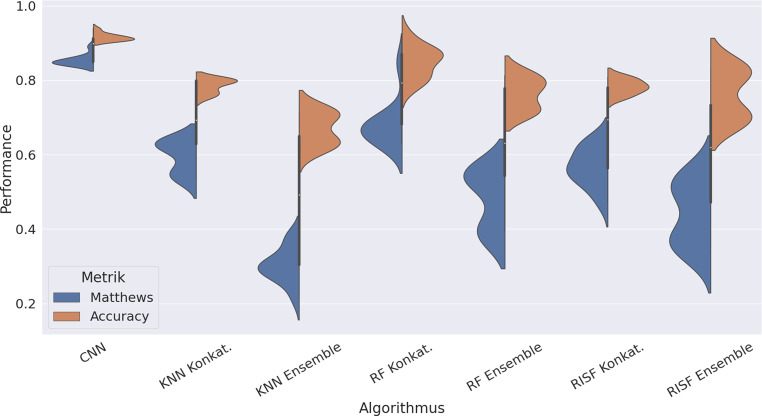

Die Darstellung der Ergebnisse erfolgt in Abb. 4 in Form eines Violinenplots, der die Verteilung des Matthews-Koeffizienten und der Genauigkeit der jeweils zehn leistungsstärksten Hyperparameter-Konfigurationen visualisiert. Zu erkennen ist dabei, dass die Genauigkeit auf Grund des Ungleichgewichts der Klassen deutlich optimistischer ausfällt als der Matthews-Koeffizient. Insbesondere bei den beiden Zeitreihen-KNN ist die größte Diskrepanz zwischen den Metriken zu beobachten, was auf eine Überanpassung an die Majoritätsklasse hinweist. Im Bereich der Zeitreihen-basierten Ansätze fällt des Weiteren auf, dass die Varianten mittels Konkatenation der Zeitreihen eine bessere Güte aufweisen als über Ensemble-Bildung. Hier sticht insbesondere der Random Forest heraus. Den performantesten Ansatz stellt jedoch das CNN dar. Hier lag das Maximum bei einem Matthews-Koeffizienten von 0,89 mit einer zugehörigen Genauigkeit von 0,94, was zugleich das Maximum der Evaluierung darstellt. Hier fällt zudem die geringe Streuung der Ergebnisse auf.

Bezüglich der betrachteten Fenstergröße lässt sich modellübergreifend feststellen, dass Zeitfenster nahe dem untersuchten Maximum von zehn Minuten aufgrund des größeren Informationsgehalts eines Samples zu besseren Klassifikationsergebnissen führen. Die Schrittgröße verhält sich antiproportional dazu. Je kleiner die Schrittweite, desto mehr Samples ergeben sich aus einem Zeitbereich, was zu einem größeren Datenbestand und damit zu einer erhöhten Stabilität des Trainings führt.

## Diskussion

In der Logistik, am Beispiel von Berufen in der Lagerwirtschaft, in Post- und Zustelldiensten sowie im Güter- bzw. Warenumschlag, wird deutlich, dass die Beschäftigten häufig sowohl mit psychischen als auch mit physischen Arbeitsanforderungen konfrontiert sind, die Fehlbeanspruchungen nach sich ziehen können und langfristig die Arbeitsfähigkeit oder Gesundheit beeinträchtigen. Trotz der permanenten Wandlungsanforderungen in der Logistik dominieren in dieser Branche vorwiegend starre Arbeitszeit- und Pausenmodelle angesichts der Tatsache, dass unterdurchschnittlich wenig Beschäftigte ihre Arbeitsabläufe sowie Arbeits- und Pausenzeiten flexibel verantworten können (Certa und Schröder 2020).

Im Themenfeld „Arbeitszeit“ repräsentiert die Pause eine arbeitsbezogene Ressource, die zu einer günstigeren Ausprägung von psychischen Gesundheitsindikatoren führen kann (BAuA 2020). Arbeitspausen haben u. a. die Funktion, beeinträchtigende Beanspruchungsfolgen auszugleichen und abzubauen. Vor allem Kurzpausen während der Arbeitszeit sowie der Faktor der Zeitsouveränität, d. h. die Möglichkeit, selbst Einfluss auf die Lage von Zeitenspannen nehmen zu können, stehen in einem positiven Zusammenhang zu verschiedenen Aspekten der psychischen Gesundheit (BAuA 2020).

Die „Dynamische Pause“ stellt einen Vorstoß dar, Stress präventiv vorzubeugen, indem die Ressource „Pause“ mit Hilfe von modernen Technologien gesundheitsförderlich eingesetzt wird. Um dies human zu gestalten und den Menschen nicht zu entmündigen, sondern Entscheidungsfreiheit auf Basis valider, privater Informationen zu gewährleisten, bedarf es zunächst der individuellen Aufnahme und Interpretation vitaler Parameter. Maschinelles Lernen ist dabei von zukunftsträchtiger Bedeutung – die Technologie ist im Stande, Muster in großen und komplexen Datensätzen zu erkennen, welche der menschlichen Auffassungsgabe verwehrt bleiben. Die Komplexität von Datenerfassung und Verarbeitung spiegelt sich vor allem in der starken Subjektivität des Stressempfindens wider, welche zudem unterbewusst oder unbemerkt stattfinden kann.

Die Ergebnisse der ML-Evaluierung haben gezeigt, dass eine Klassifikation der Beanspruchung auf Basis der objektiven Vitalparameter praktikabel scheint. Insbesondere die hohe Korrelation zwischen der Ausgabe der Modelle und des tatsächlichen Ergebnisses lassen auf eine erfolgreiche, diskriminative Abbildung schließen.

## Limitationen

Eine KI-basierte dynamische Pausenregelung kann eine wertvolle Unterstützung für den Arbeitsalltag in belastungsintensiven Berufen bieten, birgt jedoch neben der Auswahl und des Tunings des verwendeten Modells weitere Herausforderungen. Die durch künstliches Induzieren und Messen erzeugten Stressdaten der Studie treffen in der Realität möglicherweise auf die unumstößliche Individualität der Personen in einer realen Arbeitssituation mit realen mentalen und physischen Aufgaben. Die Daten in der Praxis könnten massiv von den Daten abweichen, mit denen das KI-Modell trainiert wurde und somit ungenaue oder falsche Stresssituationen erkennen. Die Technologie hat jedoch auch Potenziale, die im Laufe der Entwicklung der „Dynamischen Pause“ zu Tage gekommen sind. Ein Beispiel dafür ist die in der Studie gemessene Herzratenvariabilität. Sie zeigt eine eindeutige Veränderung der Aktivität an, jedoch ist nicht immer eindeutig, ob diese auf eine positive Aktivierung zurückzuführen ist oder negativem Stress entsprechen würde. Erste Analysen der subjektiven Fragebogenergebnisse geben Hinweise darauf, dass sich die Probanden in Folge der körperlichen Aufgaben eher positiv aktiviert gefühlt haben. Die künstliche Intelligenz könnte in diesem Zusammenhang sehr nützlich sein, da sie durchaus über die Kapazitäten verfügt, Unterschiede zwischen positiver und negativer Aktivierung zu erkennen.

Weiterhin sollen Schwächen der Datenverarbeitung in der fortschreitenden Entwicklung der „Dynamischen Pause“ aus mehreren Richtungen begegnet werden. Die subjektiven Items, die in der Studie ermittelt wurden, könnten als weitere Anhaltspunkte dienen, um die Genauigkeit des Modells zu erhöhen. Die Herausforderung an der Stelle ist, dass es aktuell an einer umfassenden Methode fehlt, subjektiven Stress abzubilden. In der Laborstudie wurden daher verschiedene Fragebögen ausgewählt, die sich den verschiedenen Aspekten und Symptomen des Stressempfindens über unterschiedliche thematische Konzepte annähern. Es ist daher dennoch davon auszugehen, dass subjektiver Stress nur näherungsweise operationalisiert werden konnte. Darüber hinaus ist eine Feldstudie mit Lagerarbeiter/‑innen aus Logistikunternehmen geplant, um weitere Daten, diesmal unter Realbedingungen, zu erzeugen. Ebenfalls ist die Evaluation einer längeren Belastungsexposition von Beschäftigten und die damit verbundene Stresswahrnehmung angedacht. Ein weiterer limitierender Aspekt ist die Datensicherheit im Arbeitskontext. Vitaldaten gehören zu den sensibelsten und persönlichsten Daten, die von einer Person erhoben werden können. Parallel zur Entwicklung der eigentlichen Anwendung wird ein umfangreiches Datenschutzkonzept entwickelt, welches die nutzende Person vor Datenmissbrauch schützen soll.

## Implikationen

Der Mensch stellt nach wie vor die wichtigste Ressource in der Logistikbranche dar und die zahlreichen Studien zur Erkennung menschlicher Vitalparameter und Gemütszustände, vor allem durch die Nutzung künstlicher Intelligenz und maschinellem Lernen zeigt einen Trend in Richtung einer engeren Interaktion von Menschen und Maschine. Die „Dynamische Pause“ kann als eine technikbasierte verhaltens- und verhältnispräventive Interventionsmaßnahme in Betrieben eingesetzt werden (Faller 2010). Im Rahmen der betrieblichen Prävention stellen kurze Erholungspausen eine primärpräventive Möglichkeit dar, längere Belastungsexpositionen zu unterbrechen, Stress damit rechtzeitig vorzubeugen und die Gesundheit der Mitarbeitenden langfristig zu erhalten. Daneben werden die Beschäftigten im Sinne der betrieblichen Gesundheitsförderung befähigt, selbstorganisiert kurze Erholungspausen einzulegen und somit aktiv bei der Gestaltung der Lebenswelt Betrieb teilzuhaben. Auf lange Sicht hat der Dienst „Dynamische Pause“ zum Ziel, die Arbeitszeitorganisation in Unternehmen gleichermaßen an die unternehmensinternen Prozesse und Anforderungen der Logistik sowie an die Bedürfnisse der Beschäftigten anzupassen. Neben einer Integration in den innerbetrieblichen Materiafluss auf IT-Ebene sollen im nächsten Schritt auch Pauseninhalt und -ort individuell und in Abhängigkeit der Belastungsexposition empfohlen werden. Die „Dynamische Pause“ besitzt somit das Potenzial, als unternehmerisches Ziel in das betriebliche Gesundheitsmanagement verankert zu werden.

Letztlich ist allerdings die Menschlichkeit selbst als die finale Hürde zu betrachten. In einer realen Implementierung besteht möglicherweise ein Akzeptanzproblem, Mitarbeitende könnten sich trotz Datenschutzkonzept überwachter als bereits, z. B. in Folge zu erfüllender Kennzahlen, fühlen. Das fehlende Vertrauen in die Sicherheit und Verwendung der eigenen Daten kann zu zusätzlichem Leistungsdruck der Arbeitenden führen und möglicherweise einen weiteren Stressor im Arbeitsalltag bilden. Vor diesem Hintergrund wird bei der Einführung und Anwendung der „Dynamischen Pause“ in der betrieblichen Praxis empfohlen, dass sich Arbeitgeber und Betriebsrat abstimmen und eine Betriebsvereinbarung aufsetzen, in der die Zweckbestimmung der „Dynamischen Pause“ benannt sein sollte. Der Betriebsrat hat zudem ein Mitbestimmungsrecht bei der Pausenorganisation und kann den mitarbeiterorientierten Einsatz der Software gewährleisten mit Blick auf Arbeitssicherheit und Gesundheitsschutz.
